# Feeding difficulty is the dominant feature in 12 Chinese newborns with *CHD7* pathogenic variants

**DOI:** 10.1186/s12881-019-0813-z

**Published:** 2019-05-30

**Authors:** Xiang Chen, Kai Yan, Yanyan Gao, Huijun Wang, Guoqiang Chen, Bingbing Wu, Qian Qin, Lin Yang, Wenhao Zhou

**Affiliations:** 1 Departments of Neonatology, Children’s Hospital of Fudan University, Shanghai, 201102 China; 2 Ultrasonography Unit, Children’s Hospital of Fudan University, Shanghai, 201102 China; 3 Shanghai Key Laboratory of Birth Defects, The Translational Medicine Center of Children Development and Disease of Fudan University, Children’s Hospital of Fudan University, 399 Wanyuan Road, Shanghai, 201102 China; 4 Departments of Endocrinology, Children’s Hospital of Fudan University, 399 Wanyuan Road, Shanghai, 201102 China

**Keywords:** CHARGE syndrome, *CHD7* gene, Variant, Feeding difficulty, Newborn

## Abstract

**Background:**

CHARGE syndrome is characterized by coloboma, heart defects, choanal atresia, growth retardation, genitourinary malformation and ear abnormalities. The chromodomain helicase DNA-binding protein 7 (*CHD7*) gene is the major cause of CHARGE syndrome and is inherited in an autosomal dominant manner. Currently, the phenotype spectrum of CHARGE syndrome in neonatal population remain elusive. We aimed to investigate the phenotype spectrum of neonatal patients suspected to have CHARGE syndrome with pathogenic or likely pathogenic variants in the *CHD7* gene.

**Methods:**

We pooled next-generation sequencing data from the Neonatal Birth Defects Cohort (NBDC, ClinicalTrials.gov Identifier: NCT02551081) in Children’s Hospital of Fudan University. The pathogenicity of novel variants was analyzed by bioinformatic and genetic analyses. Clinical information collection, Sanger sequencing and follow-up interviews were performed when possible. Cranial MRI of these patients was performed, the volumes of different regions of the brain were analyzed.

**Results:**

A total of 12 unrelated patients in our cohort were found with *CHD7* variants. Eight patients received a firm clinical diagnosis of CHARGE syndrome (Bergmann criteria, Blake criteria, Verloes criteria and Hale criteria). Three patients did not match any diagnostic criteria, and no patients matched the Verloes criteria. Phenotype spectrum analysis found that feeding difficulty was the dominant feature among this neonatal cohort. Six *novel* variants in the *CHD7* gene (Glu2408*, Lys651*, c.5607 + 1G > T, Leu373Val, Lys2005Asnfs*37 and Gln1991*) were identified, expanding the variant database of the *CHD7* gene. Cranial MRI analysis revealed significant volume loss in cingulate gyrus, occipital lobe, and cerebellum and volume gain in the left medial and inferior temporal gyri anterior white matter parts.

**Conclusions:**

Based on a relatively unbiased neonatal cohort, we concluded that CHARGE syndrome and *CHD7* gene variants should be suspected in newborns who have feeding difficulty, and one or more malformations.

**Trial registration:**

Neonatal Birth Defects Cohort (NBDC, ClinicalTrials.gov identifier: NCT02551081).

**Electronic supplementary material:**

The online version of this article (10.1186/s12881-019-0813-z) contains supplementary material, which is available to authorized users.

## Background

CHARGE syndrome ([MIM:214800]) is a rare disease with a prevalence between 1:15,000 and 1:17,000. This syndrome is mainly characterized by coloboma, heart defects, choanal atresia, growth retardation, genitourinary malformation and ear abnormalities [1]. Minor manifestations include a distinctive face, esophagus malformation, hypothalamo-hypophyseal dysfunction and mental retardation [2, 3]. Clinical diagnostic criteria were proposed by Blake in 1998 [2] and revised by Verloes in 2005 [3]. The *CHD7* gene is the only known gene [4] responsible for 90%~ 95% of typical CHARGE syndrome [1]. CHD is an acronym for chromodomian helicase DNA-binding proteins. CHD7 belongs to the CHD protein subfamily III, along with CHD5, CHD6, CHD8, CHD9 [5, 6]. CHD7 acts as an ATP-dependent chromatin remodeler and transcriptional regulator preferring short linker DNA [7]. In mouse models, *CHD7* participates in embryonic stem cell differentiation and regulates the transcription of tissue-specific genes [8]. The broad spectrum of CHARGE syndrome symptoms is related to the regulatory function of *CHD7* in the multipotent migratory neural crest in the embryonic period [9]. Bergmann et al. reported that a large number of patients with a *CHD7* variant do not fulfill the clinical criteria of CHARGE syndrome. Mildly affected patients may be overlooked easily. So, in 2011, they emphasized that *CHD7* analysis is helpful in the CHARGE syndrome diagnosis process and proposed a guideline for *CHD7* analysis [10]. In 2016, new CHARGE syndrome diagnostic criteria was proposed by Hale with four major criteria and seven minor criteria [11]. Major standards include pathogenic variants in the *CHD7* gene, coloboma, choanal atresia or cleft palate and ear (external, middle or inner) abnormalities. The inclusion criteria of CHARGE syndrome are two major criteria and any number of minor criteria. So genetic testing is increasingly important in CHARGE syndrome diagnostic process. In this study, we describe the phenotype spectrum of 12 neonatal patients carrying *CHD7* variants. This is the largest sample size with a focus on CHARGE syndrome in the Chinses Han population.

## Methods

### Inclusion criteria based on clinical features and genetic analysis

We pooled data from the Neonatal Birth Defects Cohort (NBDC, ClinicalTrials.gov Identifier: NCT02551081) in Children’s Hospital of Fudan University during 2016–01 to 2018–11. This data included 11,572 whole exome sequencing, 13,636 clinical exome sequencing and 1284 whole genome copy number microarray analyses. In this retrospective study, enrolled newborns met one of the following four criteria: 1) a previously established heterozygous pathogenic variant in the *CHD7* gene, with data obtained from both the public database (HGMD and ClinVar) and internal database; 2) the same amino acid change as a previously established pathogenic variant; however, different nucleotide changes were accepted; 3) a *novel* (both public database and internal database) heterozygous null variant (nonsense, frameshift, canonical +/− 1 or 2 splice sites, initiation codon, single or multiexon deletion) in *CHD7* gene; and 4) a *novel* and de novo heterozygous variant with negative family history or inherited from the affected parents. All variants were classified according to ACMG guideline [12]. Patients were excluded if pathogenic copy number variants were identified using array-based comparative genomic hybridization (arry-CGH). Patients’ information was obtained from clinical records. A clinical diagnosis of CHARGE syndrome was made based on the Bergmann criteria [10], Blake criteria [2], Verloes criteria [3] and Hale criteria [11].

### Next generation sequencing and sanger confirmation

The criteria for genetic testing were approved by ethics committees of Children’s Hospital, Fudan University (2014–107). Pretest counseling was performed by physicians. Informed consent was obtained from the patient’s parents. High-throughput sequencing was performed according to standard protocols in Clinical Laboratory Improvement Amendments (CLIA: 99D2064856) compliant sequencing laboratory in Wuxi NEXTCODE (China). Sequences were generated using the Agilent ClearSeq Inherited Disease Kit, Illumina Cluster and SBS Kit. Next generation sequencing was performed on an Illumina HiSeq 2000/2500 platform. The detected variants were confirmed using PCR and PCR-amplified DNA products were subjected to direct automated sequencing (3500XL Genetic Analyzer, Applied Biosystems) according to the manufacturer’s specifications. De novo variants were detected by parental evaluating via Sanger sequencing.

### Cranial MRI analysis

Among the 12 affected neonates, 8 had T1- and T2-weighted MRI head scans from the picture archiving and communication system (PACS) in Children’s hospital of Fudan University. We matched these affected neonates with 92 control neonates of the same corrected gestational age and excluded 4 patients because of image registration failure caused by poor image quality. All digital imaging and communications in medicine (DICOMs) were concatenated into a NIfTi volume format by dcm2niix software [13]. Then, the brain images of the neonates were extracted through FSL BET based on the T2-weighted modality followed by N4 bias correction [14]. The skull-striped T2 images were normalized to the neonate-specific T2 weighted image template at 44 weeks corrected gestational age [15] using the SyN registration method in ANTS [16]. The T2 brain template included a parcellation atlas with 87 regions of interest (ROI). The volumes of each ROI in the brain were measured by the sum of the log relative Jacobian determinant from the nonlinear deformable field of the registration. The total brain volume was used as a nuisance variable for regression, and the t statistic map was constructed from the linear regression as follows: ROI_volume ~α variants+ β corrected_age + θ gender+ 1.

## Results

### Clinical diagnosis

A total of 12 pathogenic/likely pathogenic variants in the *CHD7* gene were identified by next generation sequencing in 12 unrelated newborns. All enrolled patients were from nonconsanguineous couples from the Chinese Han population. All patients’ family histories were negative. The clinical manifestations of each patient are shown in Table 1. The Bergmann criteria focuses on patients with suspected features of CHARGE syndrome with *CHD7* analysis. According to Bergmann’s criteria, Patient 2, 6, 7, 8, 9, 10, 11, and 12 matched the guidelines and needed further *CHD7* analysis. Patients 7, 8 and 11 matched two cardinal and one supportive criteria and need *CHD7* analysis including MLPA. Patient 2, 6, 9 and 10 matched one cardinal criteria and one or more than one supportive criteria. According to Bergmann’s criteria, the four patients needed a temporal bone CT first to detect typical semicircular canal abnormalities. The Blake criteria has four major criteria (coloboma, choanal atresia/stenosis, characteristic ear anomalies and cranial nerve dysfunction) and seven minor criteria. Definitive CHARGE syndrome should match 4 major or 3 major features and 3 minor features. Probable or possible CHARGE is defined as 1 or 2 major features and several minor features. Based on the Blake criteria, 8 patients (2, 3, 6, 7, 8, 10, 11 and 12) were diagnosed with probable or possible CHARGE. The major criteria of Hale proposed in 2016 were coloboma, choanal atresia or cleft palate, ear abnormalities and pathogenic *CHD7* variants. Patients matching two major criteria and any number of minor criteria are diagnosed with CHARGE syndrome. In this study, four patients (2, 3, 7 and 11) met Hale’s criteria for CHARGE syndrome. Major Verlos criteria include coloboma, choanal atresia/stenosis and hypoplasia/aplasia of the semicircular canals. Patient matching as least two major criteria can be considered to have CHARGE syndrome. As no patient in our cohort was reported with choanal atresia/stenosis and abnormalities of the semicircular canals, no patient could be diagnosed with CHARGE syndrome based on the Verloes criteria. In this population, some typical features of CHARGE syndrome were observed, including aplasia/dysplasia of the semicircular canals, cleft lip/palate and choanal atresia.

**Table 1:** Phenotype spectrum of 12 neonatal patients

Item	Positive observed	Bergman 2011 [10]	1	2	3	4	5	6	7	8	9	10	11	12
Sex	75% (9/12 male)	/	M	M	M	F	M	M	M	F	M	M	F	M
GA (week)	37.54 (average)	/	41 + 2	36 + 3	NA	38 + 3	37 + 2	35 + 6	39	40	36 + 2	34 + 3	38	39 + 4
Weight (Kg)	2.77 (average)	/	3.2	3.45	NA	2.2	2.76	2.2	3.75	2.8	2.4	1.85	2.65	3.2
Age of onset	1.83 (average)	/	IAB	9d	10d	IAB	IAB	3d	IAB	IAB	IAB	IAB	IAB	IAB
Head and Neck	83% (10/12)	/												
Abnormality of the external ear	50% (6/12)	97.0% (224/231)	N	yes	yes	N	N	yes, L	yes	N	N	N	yes, R	yes, B
Abnormal location of ears	17% (2/12)	/	N	N	N	N	N	N	N	N	yes	N	N	yes
Colobomas	17% (2/12)	80.8% (189/234)	N	N	N	N	N	N	N	yes	N	yes	N	N
Hypertelorism	8% (1/12)	/	N	N	N	N	N	N	yes	N	N	N	N	N
Asymmetric crying face	33% (4/12)	/	N	N	yes	N	N	N	yes	yes	N	N	yes	N
Flat nasal bridge	8% (1/12)	/	N	N	N	N	N	N	yes	N	N	N	N	N
Micrognathia	17% (2/12)	/	N	N	N	N	N	N	N	N	N	N	yes	yes
Short neck	8% (1/12)	/	N	N	N	N	N	N	N	N	N	yes	N	N
Head circumference	33.45 (average)	/	33	36	NA	33	32	NA	35	35	33	30.5	33	34
SD (standard deviation)	/	/	(−1.37 SD)	(−0.60 SD)		(−1.22 SD)	(−1.71 SD)		(−0.40 SD)	(−1.56 SD)	(−1.27 SD)	(−2.47 SD)	(−1.22 SD)	(−1.59 SD)
Cardiovascular system	58% (7/12)	75.8% (191/252)												
PDA	100% (12/12)	/	yes	yes	yes	yes	yes	yes	yes	yes	yes	yes	yes	yes
Atrial septal defect	33% (4/12)	/	N	yes	N	N	N	N	N	yes	N	yes	yes	N
Ventricular septal defect	8% (1/12)	/	N	N	N	N	N	N	N	N	yes	N	N	N
Pulmonary valve stenosis	8% (1/12)	/	N	N	N	N	N	N	N	N	yes	N	N	N
Right aortic arch	17% (2/12)	/	yes	N	N	N	N	yes	N	N	N	N	N	N
Coarctation of aorta	17% (2/12)	/	N	N	N	N	N	N	N	yes	N	yes	N	N
Respiratory system	17% (2/12)													
Tracheal stenosis	8% (1/12)	/	N	N	N	N	N	N	N	yes	N	N	N	N
Laryngeal obstruction	8% (1/12)	/	N	N	N	N	N	N	N	N	N	N	yes (II)	N
Hypoplasia of Laryngeal cartilage	8% (1/12)	/	N	N	N	N	N	N	N	yes	N	N	N	N
Digestive system	67% (8/12)	/												
Tracheo-oesophageal malformation	35% (3/12)	28.8% (42/146)	yes	N	N	yes	N	N	yes	N	N	N	N	N
Hiatal hernia	8% (1/12)	/	N	N	N	N	N	N	N	N	N	N	N	yes
Feeding difficulty	58% (7/12)	/	yes	yes	N	yes	N	yes	yes	yes	N	N	yes	N
Urogenital system	67% (6/9)	81.4% (118/145)												
Cryptorchidism	44% (4/9)	/	N	N	N	/	N	yes, R	yes	/	yes, B	yes, B	/	N
Micropenis	33% (3/9)	/	N	N	N	/	N	N	yes	/	N	yes	/	yes
Hypospadias	11% (1/9)	/	N	N	N	/	N	N	yes	/	N	N	/	N
Small scrotum	11% (1/9)	/	N	N	N	/	yes, B	N	N	/	N	N	/	N
Limbs	17% (2/12)	/												
Polydactyly	8% (1/12)	/	N	yes, LH	N	N	N	N	N	N	N	N	N	N
Syndactyly	8% (1/12)	/	N	N	N	N	N	N	N	yes, LF, 4–5	N	N	N	N
Immudeficiency	8% (1/12)	/	NA	NA	NA	NA	NA	NA	NA	NA	NA	NA	NA	yes
Diagnosis	75% (9/12)	/												
Bergmann	67% (8/12)	/	N	yes	N	N	N	yes	yes	yes	yes	yes	yes	yes
Blake	67% (8/12)	/	N	yes	yes	N	N	yes	yes	yes	N	yes	yes	yes
Verlos	0	/	N	N	N	N	N	N	N	N	N	N	N	N
Hale	42% (5/12)	/	N	yes	yes	N	N	N	yes	yes	N	N	yes	N
Death	33% (4/12)	/	N	N	yes	N	N	N	yes	yes	N	yes	N	N

*B* bilateral, *Blank* patient without this feature, *F* female, *GA* gestational age, *IAB* immediately after birth, *L* left, *LF* left food, *LH* left hand, *M* male, *N* do not have this feature, *NA* the missing value, *PDA* patent ductus arteriosus, *R* right, *T* term baby

### Variants of CHD7

Twelve pathogenic/likely pathogenic variants in the *CHD7* gene were identified. We detected 6 reported pathogenic variants and 6 *novel* variants, including 4 frameshifts, 4 stop-gain, 2 splice-donor region, 1 intron variant and 1 missense variant (Table 2). Stop-gain and frameshift variants accounted for 67% of variants in this study. None of these variants are included in the gnomeAD database (http:// gnomad.broadinstitute.org/), the 1000 gnommeAD database (http://gnomad-old.broadinstitute.org/) or our internal database (1833 probands and 6893 families). Among all six novel variants, Glu2408* and Lys651* (NM_017780) were identified as de novo variants by Sanger sequencing. We mapped the 12 variants into the structure of the CHD7 protein (http://www.ebi.ac.uk/interpro/). Two variants were located in the chromodomain, two were located in the SANT domain, one in the ATP-binding domain belongs to the Helicase superfamily (Helicase N domain) and one was in the BRK domain. We did not detect *CHD7* copy number variants in our study.

**Table 2:** Genotype of Patients

NO.	Region	Nucleotide Change	Amino Acid Change	Type of Variants	Familial Targeted Variants Study	gnomAD/1000gene	Score of S/PP/MT	S/PP/MT	References	Classification
1	exon12	c.3082A > G	p.Ile1028Val	missense	/	0/0	0/0.894/1	D/PD/D	PMID 15300250	P
2	exon31	c.6165_6166del	p.Tyr2056Profs*3	frameshift	/	0/0	/	/	PMID 22461308	P
3	intron 25	c.5405-17G > A	/	intron	De novo	0/0	/	/	PMID 16155193	P
4	exon8	c.2504_2508del	p.Tyr835Serfs*14	frameshift	/	0/0	/	/	PMID 16155193	P
5	intron 27	c.5607 + 1G > T	/	splice_donor	/	0/0	/	/	/	LP
6	exon34	c.7222G > T	p.Glu2408*	stop-gain	De novo	0/0	0.19/NA/1	T/NA/D	/	P
7	intron 11	c.2957 + 5G > A	/	splice_donor	/	0/0	/	/	PMID 22461308	P
8	exon3	c.1951_1952delinsT	p.Lys651*	stop-gain	De novo	0/0	/	/	/	P
9	exon37	c.8062dupA	p.Ile2688Asnfs*3	frameshift	/	0/0	/	/	/	LP
10	exon30	c.6015_6018delAAAA	p.Lys2005Asnfs*37	frameshift	/	0/0	/	/	/	LP
11	exon8	c.2572C > T	p.Arg858*	stop-gain	De novo	0/0	0.1/N/1	T/N/D	PMID 16155193	P
12	exon30	c.5971C > T	p.Gln1991*	stop-gain	/	0/0	0.11/N/1	T/N/D	/	LP

*D* deleterious, *MT* MutationTaster, *N* normal, *NA* none, *LP* likely pathogenic, *P* pathogenic, *PD* probably damaging, *PMID* PubMed ID number, *PP* Polyphen2, *S* SIFT, *T* tolerant

### Cranial MRI analysis results

**Fig. 1:**
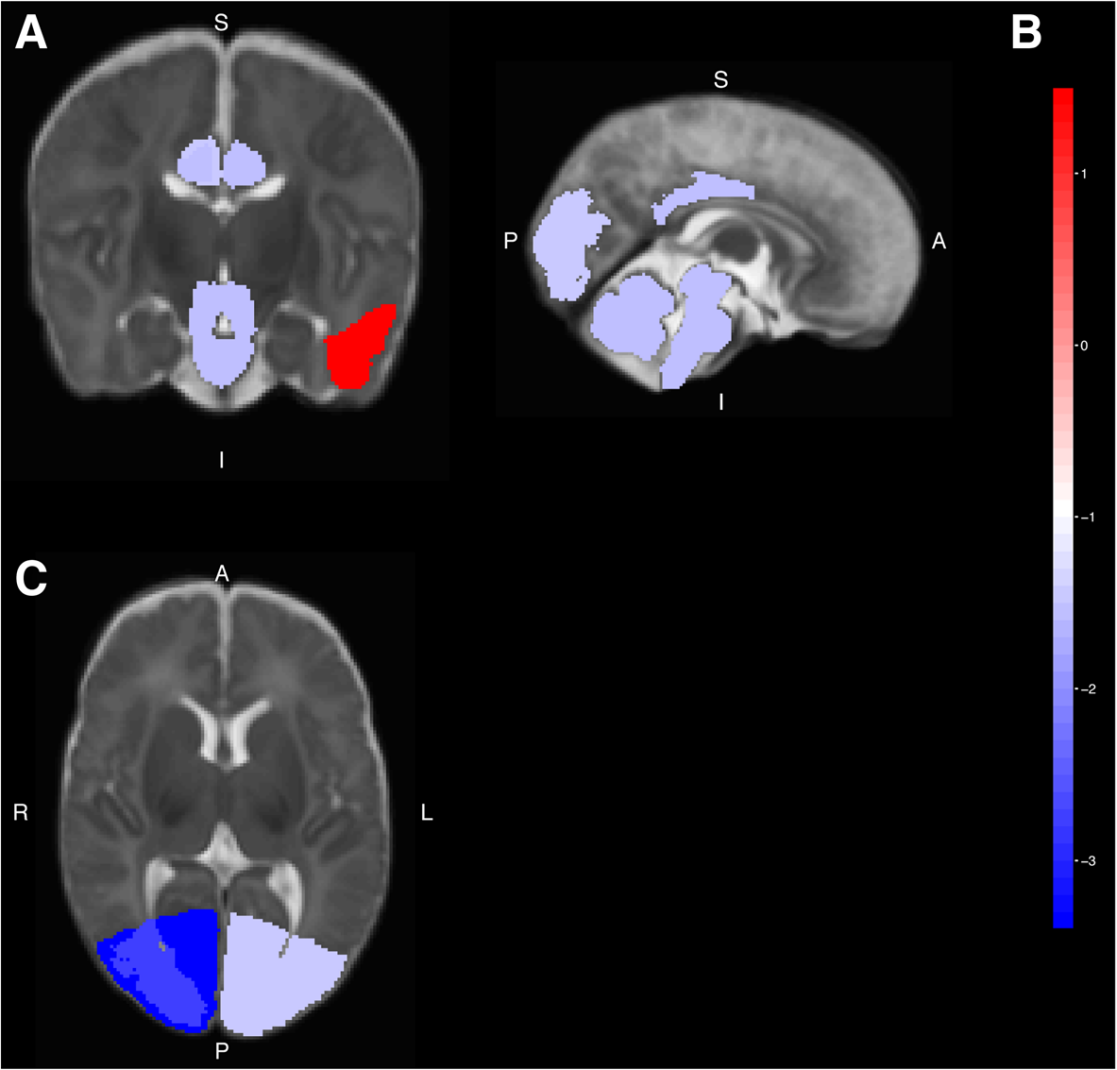
Brain volume change of patients with pathogenic/likely pathogenic variants in *CHD7*. Red color means volume gain and blue color means volume loss (*p* < 0.05). (**a**) Coronal, (**b**) Sagittal, (**c**) Axial, R: Right; L: Left; A: Anterior; P: Posterior; I: Inferior; S: Superior

MRI analysis revealed significant volume loss in the cingulate gyrus, occipital lobe, and cerebellum and volume gain in the left medial and inferior temporal gyri anterior white matter parts among neonates with the *CHD7* variant (Fig. 1). In addition, different subregions showed different levels of volume loss among these three regions (Table 3).

**Table 3:** Volume loss regions of CHARGE syndrome patients

Region of Brain	Volume loss	*p*-value
atlas50_17_Cerebellum left	−0.20947782	0.027094616
atlas50_18_Cerebellum right	− 0.229208438	0.016167437
atlas50_22_Occipital lobe right	−0.341721802	0.000452598
atlas50_23_Occipital lobe left	−0.210090999	0.037293534
atlas50_34_Cingulate gyrus, posterior part right	−0.219671229	0.028380689
atlas50_35_Cingulate gyrus, posterior part left	−0.220531078	0.029805553
atlas7_6_Cerebellum + Brainstem	−0.20603902	0.026648778
atlas87_22_Occipital lobe right GM	−0.370674616	0.000147048
atlas87_23_Occipital lobe left GM	−0.230148083	0.022420291
atlas87_27_Lateral occipitotemporal gyrus, gyrus fusiformis posterior part left GM	−0.192364134	0.0431107
atlas87_34_Cingulate gyrus, posterior part right GM	−0.213154535	0.034225732
atlas87_59_Medial and inferior temporal gyri anterior part left WM	0.214177329	0.03865777
atlas87_65_Occipital lobe right WM	−0.307703804	0.001591836
atlas87_77_Cingulate gyrus, posterior part right WM	−0.217167864	0.030227307
atlas87_78_Cingulate gyrus, posterior part left WM	−0.243852741	0.015847061
atlas87_84_Extra-cranial background	−0.228192206	0.0277017

## Discussion

The NGS test largely reduced the turnaround time of high-sequencing genetic tests; therefore, genetic diseases can be rapidly diagnosed in neonatal patients. To the best of our knowledge, this is the first study to investigate the phenotype spectrum of neonatal patients with likely pathogenic or pathogenic *CHD7* variants. According to the four reported diagnostic criteria of CHARGE syndrome, 0–67% of newborns received a clinical diagnosis. This may be because some typical features of CHARGE syndrome (aplasia/dysplasia of the semicircular canals, cleft lip/palate and choanal atresia) are not observed in this population, and some features present later in childhood (growth retardation and mental retardation) cannot be diagnosed in neonatal period. Proposed in 2016, pathogenic *CHD7* variant status is now a major criterion in CHARGE syndrome diagnoses [11]. Criteria focusing on typical clinical phenotypes may exclude patients with a mild phenotype in early life.

Digestive and respiratory problems are the primary causes of postneonatal demise in CHARGE syndrome [17]. Complex digestive anomalies in patients with CHARGE syndrome are often highly prevalent at birth and require long-term management [18, 19]. In this study, 67% (8/12) of patients had digestive system anomalies, including feeding difficulty, tracheo-oesophageal malformation and hiatal hernia. Tracey Allen et al. [20] reported that tracheo-oesophageal malformation was regarded as a conditional finding in neonates and a major factor influencing morbidity. Tracheoesophageal fistula was previously reported as an uncommon feature among CHARGE syndrome patients with a prevalence between 8%~ 18% [21]. However, Conny van Ravenswaaij-Arts [22] reported a higher prevalence (28.8%, 42/146). In our study, 3 of 12 (25%) patients had tracheo-oesophageal malformation. Our results showed that feeding difficulty was the main feature among neonatal patients. Therefore, the possibility of CHARGE syndrome should be kept in mind when newborns suffer from feeding difficulty for a period.

Heart defects are predominant features in CHARGE syndrome patients and patients with pathogenic *CHD7* variants [23]. Patent ductus arteriosus is more commonly seen in patients with CHD7 pathogenic variants than nonsyndromic heart malformations [24]. In our study, all patients had patent ductus arteriosus. As the patent ductus arteriosus can be closed within 3 months of age in most babies, the proportion of heart defects, including patent ductus arteriosus, may be overestimated in the neonatal population.

In our study, 4 neonates with CHARGE syndrome had an asymmetric crying face. To the best of our knowledge, this study first reported the asymmetric crying face in CHARGE syndrome. An asymmetric crying face is recognized in neonates as lower lip asymmetry present only with crying. The etiology includes facial nerve compression and faulty facial muscle/nerve development. An asymmetric crying face is often seen in 22q11.2 deletion syndrome patients; however, it has not been reported in CHARGE syndrome patients [25, 26]. This could be because the importance of this symptom has not received enough attention.

Respiratory distress during feeding, regurgitation and persistent frothy salivation indicate a high risk of esophageal atresia or tracheoesophageal fistulae [27]. Treatment of feeding difficulty caused by esophageal atresia needs a multidisciplinary team to manage additional specific medical or physiological problems [28]. Although the survival of these patients is as high as 90%, long-term complications are common, continuous and challenging [29]. Additionally, surviving patients diagnosed with CHARGE syndrome most likely suffer from development delays, especially in motor and language development [30].

For CHARGE syndrome patients with a *CHD7* pathogenic variant, the relationship between genotype and phenotype is unclear [10]. In this study, stop-gain, frameshift and splice variants were the main variants, which is in agreement with a previous study [31]. The patients in this study with the known pathogenic variant presented different phenotypes from the reported patients. All this indicates that CHARGE syndrome is a highly heterogeneous disease and *CHD7* gene analysis is important for comprehensive assessment.

MRI was used to detect cochlear abnormalities in CHARGE syndrome patients. Recent studies showed that *CHD7* affects neurogenesis by activating neuron stem cells and progenitors [32]. Conditional genetic deficiency of *Chd7* in mice led to abnormalities in corpus callosum and cerebellum [33, 34]. Christa M. de Geus et al. published a cranial imaging evaluation checklist for CHARGE patients based on a literature review [35]. Cerebellum dysplasia was included as a cranial abnormality, but abnormalities of the cingulate gyrus and occipital lobe were not mentioned. Therefore, our study provides evidence to expand the abnormalities presented in the cranial imaging of CHARGE patients. According to previously studies, the cingulate gyrus processed and modulated gastrointestinal sensory signals [36], and the cerebellum involved in the regulation of feeding behavior [37]. These findings may explain why feeding difficulty is the dominant feature of neonatal CHARGE patients. The relationship between the occipital lobe, temporal lobe and feeding difficulty remains unknown. As only a few cranial MRI data of neonatal patients can be used for region division, only four patients were analyzed in this study. High-quality data collection from neonatal patients will be helpful for further study.

In this study, we reported 12 neonatal patients with *CHD7* pathogenic/likely pathogenic variants. Six *novel* variants in the *CHD7* gene were identified, expanding the variant database of the *CHD7* gene. Diagnostic criteria of CHARGE syndrome focusing on only typical clinical features may underestimate its neonatal incidence. CHARGE syndrome and *CHD7* pathogenic variants should be suspected in newborns who have feeding difficulty and one or more malformations.

## Conclusions

Our study found that diagnostic criteria of CHARGE syndrome focusing on only typical clinical features may underestimate neonatal incidence. Based on a relatively unbiased neonatal cohort, we concluded that CHARGE syndrome and *CHD7* gene variants should be suspected in newborns who have feeding difficulty, and one or more malformations.

## Additional file

Additional file 1: **Table S1.** Filtered variations of 12 patients. (XLS 552 kb)

## Abbreviations

CHD: chromodomain helicase DNA-binding
CLIA: Clinical Laboratory Improvement Amendments
DICOMs: digital imaging and communications in medicine
MLPA: multiplex ligation-dependent probe amplification
NBDC: Neonatal Birth Defects Cohort
NGS: next generation sequencing
PACS: picture archiving and communication system

## References

[1] Hsu P, Ma A, Wilson M, Williams G, Curotta J, Munns CF, Mehr S (2014). CHARGE syndrome: a review. J Paediatr Child Health.

[2] Blake KD, Davenport SL, Hall BD, Hefner MA, Pagon RA, Williams MS (1998). CHARGE association: an update and review for the primary pediatrician. Clin Pediatr (Phila).

[3] Verloes A (2005). Updated diagnostic criteria for CHARGE syndrome: a proposal. Am J Med Genet A.

[4] Vissers LE, van Ravenswaaij CM, Admiraal R, Hurst JA, de Vries BB, Janssen IM (2004). Mutations in a new member of the chromodomain gene family cause CHARGE syndrome. Nat Genet.

[5] Hall JA, Georgel PT (2007). CHD proteins: a diverse family with strong ties. Biochem Cell Biol.

[6] Marfella CG, Imbalzano AN (2007). The Chd family of chromatin remodelers. Mutat Res.

[7] Manning BJ, Yusufzai T (2017). The ATP-dependent chromatin remodeling enzymes CHD6, CHD7, and CHD8 exhibit distinct nucleosome binding and remodeling activities. J Biol Chem.

[8] Schnetz MP, Bartels CF, Shastri K, Balasubramanian D, Zentner GE, Balaji R (2009). Genomic distribution of CHD7 on chromatin tracks H3K4 methylation patterns. Genome Res.

[9] Bajpai R, Chen DA, Rada-Iglesias A, Zhang J, Xiong Y, Helms J (2010). CHD7 cooperates with PBAF to control multipotent neural crest formation. Nature.

[10] Bergman JE, Janssen N, Hoefsloot LH, Jongmans MC, Hofstra RM, van Ravenswaaij-Arts CM (2011). CHD7 mutations and CHARGE syndrome: the clinical implications of an expanding phenotype. J Med Genet.

[11] Hale CL, Niederriter AN, Green GE, Martin DM (2016). Atypical phenotypes associated with pathogenic CHD7 variants and a proposal for broadening CHARGE syndrome clinical diagnostic criteria. Am J Med Genet A.

[12] Richards S, Aziz N, Bale S, Bick D, Das S, Gastier-Foster J (2015). Standards and guidelines for the interpretation of sequence variants: a joint consensus recommendation of the American College of Medical Genetics and Genomics and the Association for Molecular Pathology. Genet Med.

[13] Li X, Morgan PS, Ashburner J, Smith J, Rorden C (2016). The first step for neuroimaging data analysis: DICOM to NIfTI conversion. J Neurosci Methods.

[14] Tustison NJ, Avants BB, Cook PA, Zheng Y, Egan A, Yushkevich PA, Gee JC (2010). N4ITK: improved N3 bias correction. IEEE Trans Med Imaging.

[15] Oishi K, Mori S, Donohue PK, Ernst T, Anderson L, Buchthal S (2011). Multi-contrast human neonatal brain atlas: application to normal neonate development analysis. Neuroimage.

[16] Avants BB, Epstein CL, Grossman M, Gee JC (2008). Symmetric diffeomorphic image registration with cross-correlation: evaluating automated labeling of elderly and neurodegenerative brain. Med Image Anal.

[17] Bergman JE, Blake KD, Bakker MK, du Marchie SG, Free RH, Van Ravenswaaij-Arts CM (2010). Death in CHARGE syndrome after the neonatal period. Clin Genet.

[18] Hudson A, Macdonald M, Friedman JN, Blake K. CHARGE syndrome gastrointestinal involvement: from mouth to anus. Clin Genet. 2016. 10.1111/cge.12892 28155231

[19] Dobbelsteyn C, Peacocke SD, Blake K, Crist W, Rashid M (2008). Feeding difficulties in children with CHARGE syndrome: prevalence, risk factors, and prognosis. Dysphagia.

[20] Allen Tracey (2012). CHARGE Syndrome. Advances in Neonatal Care.

[21] Lalani SR, Safiullah AM, Fernbach SD, Harutyunyan KG, Thaller C, Peterson LE (2006). Spectrum of CHD7 mutations in 110 individuals with CHARGE syndrome and genotype-phenotype correlation. Am J Hum Genet.

[22] van Ravenswaaij-Arts C, Martin DM (2017). New insights and advances in CHARGE syndrome: diagnosis, etiologies, treatments, and research discoveries. Am J Med Genet C Semin Med Genet.

[23] Corsten-Janssen N, Scambler PJ (2017). Clinical and molecular effects of CHD7 in the heart. Am J Med Genet C Semin Med Genet.

[24] Corsten-Janssen N, Kerstjens-Frederikse WS, du Marchie SG, Baardman ME, Bakker MK, Bergman JE (2013). The cardiac phenotype in patients with a CHD7 mutation. Circ Cardiovasc Genet.

[25] Pasick C, McDonald-McGinn DM, Simbolon C, Low D, Zackai E, Jackson O (2013). Asymmetric crying facies in the 22q11.2 deletion syndrome: implications for future screening. Clin Pediatr (Phila).

[26] Liang X, He B (2018). Congenital asymmetric crying facies syndrome: a case report. Medicine (Baltimore).

[27] Aminde LN, Ebenye VN, Arrey WT, Takah NF, Awungafac G (2014). Oesophageal atresia with tracheo-oesophageal fistula in a preterm neonate in Limbe, Cameroon: case report & brief literature review. BMC Res Notes.

[28] Ramsay M, Birnbaum R (2013). Feeding difficulties in children with esophageal atresia: treatment by a multidisciplinary team. Dis Esophagus.

[29] Smith N (2014). Oesophageal atresia and tracheo-oesophageal fistula. Early Hum Dev.

[30] Lalani SR, Hefner MA, Belmont JW, Davenport S (1993). CHARGE Syndrome.

[31] Janssen N, Bergman JE, Swertz MA, Tranebjaerg L, Lodahl M, Schoots J (2012). Mutation update on the CHD7 gene involved in CHARGE syndrome. Hum Mutat.

[32] Feng W, Khan MA, Bellvis P, Zhu Z, Bernhardt O, Herold-Mende C, Liu HK (2013). The chromatin remodeler CHD7 regulates adult neurogenesis via activation of SoxC transcription factors. Cell Stem Cell.

[33] He D, Marie C, Zhao C, Kim B, Wang J, Deng Y (2016). Chd7 cooperates with Sox10 and regulates the onset of CNS myelination and remyelination. Nat Neurosci.

[34] Feng W, Kawauchi D, Korkel-Qu H, Deng H, Serger E, Sieber L (2017). Chd7 is indispensable for mammalian brain development through activation of a neuronal differentiation programme. Nat Commun.

[35] de Geus CM, Free RH, Verbist BM, Sival DA, Blake KD, Meiners LC, van Ravenswaaij-Arts C (2017). Guidelines in CHARGE syndrome and the missing link: cranial imaging. Am J Med Genet C Semin Med Genet.

[36] Lawal A, Kern M, Sanjeevi A, Antonik S, Mepani R, Rittmann T (2008). Neurocognitive processing of esophageal central sensitization in the insula and cingulate gyrus. Am J Physiol Gastrointest Liver Physiol.

[37] Zhu JN, Wang JJ (2008). The cerebellum in feeding control: possible function and mechanism. Cell Mol Neurobiol.

